# Yellow Fever and the Fallacy of the Germ Theory in Connection with Its Spread and Epidemicity

**Published:** 1891-04

**Authors:** John P. Wall

**Affiliations:** Tampa, Fla.


### YELLOW FEVER AND THE FALLACY OF THE GERM THEORY IN CONNECTION WITH ITS SPREAD AND EPIDEMICITY.

By JOHN P. WALL, M. D., TAMPA, FLA.

Since the subject of bacteriology has received so much attention from the medical profession, it has naturally assumed a prominent position in the causation of disease, as an evolution of the germ theory, in explanation of the cause of fevers.

The bacillus for this and the coccus for that disease are now almost universally accepted as the pathogenic causes on the evidence simply of some *ipse dixit* who busies himself with infinitesimals through a powerful microscope and culture media. So far no practical result in the prevention or management of human ailments has been realized, while it has had a demoralizing tendency on both the profession and the general public—on the profession in inducing a theoretical practice, and on the public in misleading it to accept as proven the vague theories held by the profession of medicine on the germ theory. The general public talks about the germs or microbes of diseases with all the earnestness and credulity of proven facts ; and, as a result, have unbounded faith in disinfectants and germicides to prevent the spread of contagious and infectious diseases.

Now the truth is, very little is known with certainty about pathogenic germs ; and where micro-organisms are found in diseases, the question naturally arises as to whether such micro-organisms are the cause or the result of the disease in question. The labors of a few able but over-enthusiastic men have impressed on the majority of the profession the theories for proven truths. Time and experience have not demonstrated the teachings of Lister and Pasteur to be correct, though it is possible that their teachings have been somewhat beneficial in promoting new departures from old methods. It may be only necessary to point out the fact that the antiseptic measures of Lister to exclude atmospheric germs as a cause of suppuration, have resulted in aseptic measures based on cleanliness ; and that the so-called vaccination of Pasteur for chicken cholera and charbon have not yielded the protective results so confidently predicted and at one time believed ; and that his rabietic vaccinations do not possess the efficacy in preventing hydrophobia as claimed by him and his disciples. The discovery of the cholera bacillus by Koch has led to no practical result ; and the same may be safely predicated of his tubercule bacillus and his present experimental essays at curing consumption, as meagerly detailed in the daily press.

In view of how little is positively known in support of the germ theory—which at best can only be considered a mere hypothesis of contagious or infectious poison being a particulate matter—it is neither wise nor judicious to place such credence in it as to conform our practice in the treatment or prevention of disease to it ; in short, to accept it as a principle that all treatment or prevention of disease depends on the use of a germicide. This point is about reached by that enthusiastic majority of the profession, especially its junior members, who affect to believe the practice of medicine in the curing of disease to be a science instead of an art, as it always has been and will probably remain.

In short, the germ theory is probably as fallacious as many other theories which have sprung up in medicine, been accepted and flourished for awhile, to be finally forgotten or only remembered as curiosities of medical literature. That there may be micro-organisms developed in disease may be assumed as an indisputable fact, but instead of their being pathogenic causes, is it not more probable that the development of these micro-organisms is the result of a retrograde metamorphosis in the process of destruction—the necessary concomitant of a lowered vitality in disease ?

The idea of inoculating with attenuated germs by successive cultures or a process of more or less partial desiccation has no analogy whatever to the vaccination for smallpox, because in the latter a virus of a specific character is used, which experience alone has demonstrated to be efficacious, the same as it has that mercury is the remedy for syphilis, or that numerous other drugs produce specific effects, the rationale of which is not understood. We know that it is so, and that is all.

Finally, it may be affirmed that, even admitting the existence of specific germs of certain diseases, these germs have to be introduced into the body of another to insure their potency in producing the same disease ; and as this is most usually accomplished by contact with, or close approximation of the healthy to the sick, such diseases are known as contagious. On the other hand, infectious diseases are dependent on a general morbific agency in the atmosphere, and are not dependent on personal contact.

So much by way of preface is deemed necessary for a correct understanding of the extent of our positive knowledge in regard to germs and the germ theory, before proceeding to discuss the chief epidemic disease which occasionally prevails in the cities of the South, to-wit, yellow fever, and the most rational means to prevent its introduction and spread. It has so far failed of demonstration or proof that yellow fever is a germ disease, notwithstanding the fact that most recent authors speak of it as such. That yellow fever is occasioned by a specific poison is unquestionably a fact, and that this poison is given off by a person sick with the disease is doubtless equally true ; but this morbific emanation may be rather compared to odors which, so far, have eluded detection, if of a particulate nature, than assumed to be vital germs. Besides, my observations have convinced me that there has to be in the atmosphere of the locality the presence of some hitherto unknown factor, to render it susceptible of becoming infectious ; *i. e.*, in other words, to produce an epidemic after the introduction of the poison. What this unknown factor in the atmosphere of the locality is dependent upon is a mere matter of conjecture. We know, however, that it usually pertains to urban populations, and hence has generally been attributed to the presence of human filth. And while this may be so, I am very well satisfied that it is not always the case, but can offer no explanation other than that of Pettenkoffer, who speaks of certain localities having the "disposition" for the spread of epidemic diseases. It is a matter of observation that on the introduction into a place of a case of yellow fever, the disease does not spread by contact with unacclimated persons, and that it will generally be noticed, where the presence of the first case has been positively known, that some time, probably a week or more, will elapse before another or other cases will make their appearance, thereby clearly indicating that the poison emanating from the sick does not possess contagious properties and may prove inert in producing a general local atmospheric epidemic condition, unless there is present this unknown factor—this "disposition" of Pettenkoffer—necessary for the poison to leaven the whole, as it were. Again, another curious fact about the infection of yellow fever is the tenacity of the infection to the locality for a considerable length of time. This is observed in the case of ships, notwithstanding the fact of their departure from the infected port and sailing through the pure atmosphere of mid-ocean, where there is none of the accumulated filth of cities or towns. And this occurred, too, in 1888, in the case of the United States war vessels visiting the ports of Hayti, where it may be fairly presumed the ships were kept in a good sanitary condition, and all reasonable precautions taken to prevent their becoming infected. It is very evident, then, that it is the locality—whether a house, ship or town—which becomes infected with the poison of the disease ; and that those susceptible, if exposed in the infected locality, run the risk of contracting the disease. That there is no contagion from mere personal contact in yellow fever, as in the case of the exanthematous fevers, has been admitted by all observers of large experience with the disease, with few exceptions, for the last hundred years. And, again, in support of the view that yellow fever is not a contagious disease, it is only necessary to bear in mind the fact that season, altitude and locality even influence for or against its epidemicity—conditions that have no influence whatever on the spread of contagious diseases. Nor is there any better evidence in support of the idea of contingent or miasmatic-contagion than there is of contagion simple and pure in this disease. It may be further admitted as an established fact, that the infective principle or poison emitted from the sick with yellow fever becomes attached to goods or clothing, and retains its morbific potency for some time, the length of which is entirely dependent on its exclusion from a free airing. It is moreover highly probable that for goods or clothing to become such fomites, it is necessary for sick persons with the disease to be in the same room or building.

That epidemicity of yellow fever is dependent on a high temperature is a characteristic fact of coeval recognition with the disease itself; and even in tropical climates where the disease has become endemic, its epidemicity is only noted during the hot season of the year. In such climates the temperature never falls to the freezing point, which observation has abundantly demonstrated to be sufficient to purify the infected atmosphere of a locality; though, unless such a low temperature is continuous for some time, it may not be sufficient to destroy the infection in dwellings; and then the disease may continue in sporadic cases contracted in such infected dwellings until the following hot season, again to become epidemic if sufficient unacclimated material is in the place. And thus, too, dwellings closed and abandoned during the prevalence of an epidemic may possibly retain the infection for some time after the general infection has disappeared; but this I am inclined to doubt. The idea of the hibernation of the specific infection of yellow fever without the occurrence of sporadic cases during the winter, I feel fully convinced is a fallacy, at least in any part of the United States. And it is only in the winter season that isolation and disinfection in the management of these sporadic cases will prove of any benefit whatever. By the timely recognition and proper management of these sporadic cases during the cool season, the disease can be stamped out and its recurrence prevented the following warm season. But where the locality has the "disposition" for the spread of the infection isolation and disinfection will prove totally unavailing in preventing an epidemic during the warm season of summer and autumn, provided the disease is introduced. Of course this isolation and destruction of goods, with the use of the so-called germicides in the way of disinfection, may get the credit of having averted an epidemic in places lacking the "disposition"—the unknown necessary factor—for an epidemic spread of the disease.

As regards disinfectants it may be safely affirmed that their efficacy, like the supposed pathogenic micro-organisms for whose destruction they are used, is largely hypothetical. Even the dioxide of sulphur, which, for almost ages, has been mainly relied on for disinfecting purposes, is now acknowledged to possess no such germicidal property, and during the current year leading medical journals have slurred at it, and asked how long it will be before it is relegated to limbo by the sanitarians? Thus we see how long a delusion has held full sway over scientific (?) minds to the general satisfaction of the profession and the public. What a commentary on our boasted science! And of the many other so-called germicides that have been discovered to possess such properties since Lister first introduced carbolic acid as such, is it not probable that none of them possess any more efficacy as germicides than sulphur? Besides, so far the use of disinfectants to destroy the infection of yellow fever is purely theoretical, as no germ of the disease has been discovered, though some may have deluded themselves in the belief that they had. The use of gases for disinfecting purposes may probably do some good by displacing the stagnant atmosphere in a ship or in a room, but beyond this their utility is probably *nil*, or at least problematical. And it is a question whether or not the same end might not be attained by the introduction of pure fresh air. In fact, it is admitted that aeration is a disinfectant; and it is more than likely that the exposure of baggage and goods to the fresh air in their manipulation at quarantine stations in the process of their submission to disinfecting agents, is in truth the disinfectant after all, so far as the infection of yellow fever is concerned. In fact the exposure of baggage to air for its disinfection in case of yellow fever was suggested and recommended several years ago by Dr. Henry F. Campbell, of Augusta, Ga., in a paper published by the A. P. H. Association. And it must have occurred to every one who has witnessed the prefunctory sulphur fumigation at quarantine stations along lines of railroads during an epidemic of yellow fever, to note its farcical character and total unreliability. In 1887 a fumigating station was maintained between Tampa and Plant City from about the 10th of October till about the 20th of November, when all the time cases of yellow fever were occurring in a small hotel in Plant City, which latter place was free from communication by rail with the remainder of the State. No one sick with the disease in Plant City died till the 14th day of November, and even after that no quarantine was instituted against Plant City that year. This is mentioned as showing the absurdity of land quarantine; and many other instances of a like character within my knowledge occurred in Florida during the epidemic of 1887 and 1888. Land quarantines have been pronounced useless by all recent International Sanitary Conferences; and it does seem that the time has about arrived when health officers should begin to appreciate an expression of the collective wisdom based on experience of the civilized world; and cease to magnify their importance by countenancing the absurd and ridiculous measures of a false teaching. The general disinfection and destruction of goods, *et cetera*, practiced in Florida in 1887–'88, was nothing more nor less than a recurrence to the same absurd measures practiced in Philadelphia in 1793, which time and experience demonstrated to be worse than futile. And even in the most southern State of the Union the same fact has been most fully demonstrated under my own observation, so that it is to be hoped that no such folly will be perpetrated again under the sanction of medical authority.

And here it may not be amiss to refute the general prevalent idea obtaining in the public mind as to the great mortality from yellow fever. During an epidemic a considerable proportion of the cases are so mild as not to require the services of a physician, the disease proving to be a mere ephemeral fever of from one to three days' duration. The popular idea among people who have never passed through an epidemic that all subjects of yellow fever are violently ill and only recover by the skin of their teeth, as it were, is the main popular fallacy which has inspired such terror of the disease and occasioned such panics. The mortality of yellow fever in this country is no greater than that of other fevers, rarely exceeding ten per cent. But in consequence of an epidemic running its course in from sixty to ninety days, the number of sick and deaths in an unacclimated population is necessarily large during that period of time, though the proportion of deaths to the number of cases will rarely exceed, as before stated, ten per cent. And even this great mortality is in a great measure due to the fact that the epidemic is largely incident to the poorer classes who have not the means to follow the example of those with means who get away from the infected locality as soon as they become convinced of the true nature of the disease. Consequently many of those remaining and falling sick receive neither proper medical attention nor nursing and care. The late Dr. Bemiss estimates that the percentage of deaths in New Orleans in 1878 among those families who were able to provide medical attention and nursing did not exceed from seven to ten per cent. Besides, the numerous mild cases to which a physician may not have been called are never reported. For instance, I have repeatedly found, on being called in to see one member of a family, that several members of it had already had the disease and convalesced under domestic treatment; and that probably only one or two in a large family would be at all seriously ill. And here it may be stated that no case of yellow fever is, as a rule, serious unless it is complicated with an acute nephritis and albumen is noted in the urine by the third or fourth day, sometimes earlier than the third day in very bad cases, though not very frequently. And, moreover, it is only in cases with the nephritic complication that the icteric hue or color is developed which gives this fever its name—yellow fever.

The number of those exposed in an infected place who have the fever after leaving it is very small. According to the report on Camp Perry of Dr. Guiteras during the epidemic in Jacksonville in 1888, one thousand two hundred and eight persons were subjected to ten days' quarantine detention at that place. There were thirty-six cases of the fever during the time at Camp Perry, though two of these, already sick, were brought there from Uptonville, in Georgia, leaving thirty-four cases; and of these five sick were from Callahan and one sick from Fernandina and four sick from Jacksonville; thus reducing the number occurring among refugees proper to twenty-four—hardly two per cent. It must be remembered that Camp Perry only became a refugee quarantine station to any extent some weeks after the epidemic was in full vigor in Jacksonville. According to the same authority no one contracted the disease at Camp Perry from coming in contact with the sick, thus demonstrating and proving two important facts: First, that yellow fever is not contagious, and second, that Pettenkoffer's "disposition" in the locality for its spread was lacking. The same thing was also witnessed at the Sand-Hill Hospital, four miles from Jacksonville, where the unacclimated attendants did not contract the fever, though some of them subsequently had it in Jacksonville after a few days' sojourn in the infected atmosphere of that city. It is thus seen how few fall sick after leaving the infected locality.

To prevent the introduction of yellow fever into the Gulf and South Atlantic ports from the tropical regions south of us, a a system of inspection at the infected port to exclude from passage and transportation unacclimated persons and infected baggage and household goods, offers the most certain and satisfactory method. No matter what quarantine system is established at our own ports, such can never afford as much security as a system of exclusion at the foreign infected port. This is especially true in regard to regular passenger steamers. For mere trading vessels and steamers which have remained in the infected port for days or weeks, an inspection at a quarantine station would be necessary; and such vessels should be cleared to touch first at some government quarantine station before entering any southern port. It will always be found impossible for any quarantine station at any port to be conducted in a manner thoroughly satisfactory to remote places having communication by rapid transit with that particular seaport. Even New York's quarantine has been a subject of animadversion when cholera is prevailing in Europe; and the same would be the case in regard to yellow fever, but for the fact of that and other northern cities having been so long exempt from an epidemic of that disease.

As to preventing the spread of yellow fever from an infected city or town in the yellow fever belt of the Southern States, the problem is an extremely difficult one; and this difficulty is greatly enhanced by the terrors of the disease inspired in the public mind in latter years by the profession and the daily press. Panics are not only to be deprecated but if possible prevented by diffusing more correct ideas of the character of the disease, and disabusing the public mind as to its great mortality. Thus it is to be hoped unseemly panics may be prevented, and those wishing to leave an infected locality might be enabled to do so in an orderly way. A refuge camp a few miles from the infected city might be provided for a few days' detention of the unacclimated and the airing of baggage; though baggage from houses in which have occurred cases of fever, should not be permitted carried out of the infected locality. Airing and exposure of baggage to the hot sun for a few days would doubtless be found just as efficient in relieving articles of clothing of any slight infection contracted from the general atmospheric infection of the locality as any other means that could be devised, to say nothing of the damage to many articles of clothing by subjecting them to disinfecting processes.

Isolation of the very few who might fall sick in the refuge camp after leaving the infected locality should, of course, be provided for as a matter of precaution. But while this programme appears easy of accomplishment, its execution is surrounded with many inherent difficulties growing out of the individual independence naturally pertaining to American citizenship. Sanitary cordons are a humbug as a safeguard in preventing ingress into or egress out of the infected locality.

During the civil war yellow fever was epidemic in Charleston in 1864. In those days no such thing as a land quarantine was ever heard or thought of in this country. Troops were continually passing in and out, and one or more wayside hospitals for furloughed sick and wounded soldiers were open in the city, where the feeble not able to endure the continuous travel to their homes in other Southern States, could stop over for rest or treatment. Great numbers availed themselves of this wayside hospital for short periods of a few days to a week, and proceeded on their journeys to their homes. Communication with Savannah and other cities by rail was daily, and yet no one thought of such an absurd thing as a land quarantine. Nor did the fever prevail at any other points in the South that season. The same state of facts was experienced in Wilmington, N. C., in 1862, except as to the wayside hospitals, communication being maintained with the infected city during the prevalence of the epidemic.

Nor is there the least foundation for the belief that the disease can be contracted from the dead of yellow fever, for I remember distinctly to have seen subjects who died with the disease, on the dissecting tables of the medical college in Charleston in 1856, when I had the honor of being a student of medicine of the institution. It was even claimed by some of the writers in the first half of the present century that there was no risk of spreading the infection in a city from the introduction of the sick, provided the sick were introduced in a nude condition, deprived of all clothing; and Dr. Cartwright mentions that this method was practiced with safety in Memphis in the transportation of the sick from the New Orleans steamers to the Marine hospital. These facts are deemed important in refuting the absurd notion now obtaining to some extent, of the yellow fever infection being dormant for years in cemeteries where its victims have been buried, to be resuscitated by disturbing the graves. The hasty and often indecent burial of the dead under the false idea of speedily getting rid of an intensified infective focus, is another absurdity of the present day to be reprobated as equally repugnant to truth, human sympathy and Christian sentiment, to say nothing of the increased terrors of the disease inspired by a knowledge of such a barbarous practice, not only in the minds of the sick, but of those still well, possessing no immunity from a previous attack.

In conclusion, while acknowledging that the laws governing the origin and spread of yellow fever are unknown, it is nevertheless our duty to make the hygienic condition of towns and cities exposed to its invasion as perfect as possible in the hope of eliminating the unknown factor, the "disposition," whose presence is essential for its epidemicity. In the direction of sanitary improvement should the hope and expectation of the public be directed, rather than to a reliance on quarantine measures instituted in our own ports. And even Dr. Holt, since his retirement from the Board of Health of Louisiana, has emphatically declared that "New Orleans to be saved must be cleaned and drained," though only a few years since, as a health officer, he appeared to have perfect confidence in his system. If New Orleans can only be saved by being cleaned and drained, whence the necessity for all this furor about the Holt System ? During the quarantine season of last year a case of yellow fever got into New Orleans by way of shipping through her quarantine; but that no epidemic followed was by no means due to her efficient (?) quarantine.

When it is remembered the exceedingly small percentage of unacclimated taking sick after leaving an infected locality, and the benefits derived from the inspection of passenger steamers in Havana by a medical officer of the Marine Hospital service, it is quite probable that New Orleans' exemption from an epidemic during the last decade is rather dependent on these latter circumstances than due to her quarantine system. In fact, since the Holt system was put in operation I do not think that any cases of yellow fever have been reported at her quarantine station, or at least none arriving on the regular passenger steamers of the Morgan Line; and consequently the natural inference is that it has proven wholly unnecessary so far as passenger steamers are concerned. This, too, has been demonstrated at Tampa during the past six years.

